# Structural Study of Nano-Sized Gahnite (ZnAl_2_O_4_): From the Average to the Local Scale

**DOI:** 10.3390/nano10050824

**Published:** 2020-04-26

**Authors:** Giorgia Confalonieri, Nicola Rotiroti, Andrea Bernasconi, Monica Dapiaggi

**Affiliations:** 1 Dipartimento di Scienze della Terra, Università degli Studi di Milano, 20133 Milano, Italy; giorgia.confalonieri@unimore.it (G.C.); nicola.rotiroti@unimi.it (N.R.); andrea.bernasconi@hotmail.it (A.B.); 2 Dipartimento di Scienze Chimiche e Geologiche, Università degli Studi di Modena e Reggio Emilia, 41125 Modena, Italy; 3 Ideal Standard International, Borgo Valbelluna (BL), 32028 Belluno, Italy

**Keywords:** local structure, Zn–Al spinel, average structure, pair distribution function

## Abstract

Spinel gahnite (ZnAl_2_O_4_) has been obtained through a hydrothermal synthesis method with a grain size of about 2 nm. The sample was calcined for a few hours at two different temperatures (800 and 900 °C) in order to obtain larger grain sizes to be analyzed by means of powder diffraction with the Rietveld method, and by means of total scattering with the Pair Distribution Function (PDF) method. The idea is to compare the average to the local structure, as a function of increasing grain size. The total scattering data were collected at the European Synchrotron Radiation Facility (ESRF), Grenoble. The samples have been also characterised by means of high resolution Transmission Electron Microscopy (TEM), showing an increasing grain size up to about 9 nm. The average structure presented variations in the inversion degree and an increase in grain size. TEM observations demonstrated that the small crystals are well crystallised: the high resolution images neatly showed the atomic planes, even in the smallest particles. However, the average structure did not properly fit the PDF data in the local region, owing to a slightly different coordination among the octahedra. A new structural model is proposed for the local region of the PDF, that helped our understanding of the differences between a real nanostructured sample and that of a microcrystalline one. The oxygen disorder, due to the inversion grade of the spinel, is demonstrates to be at the basis of the local deviation. No signals of interstitial Zn atoms were detected.

## 1. Introduction

Gahnite is a spinel with a chemical formula ZnAl_2_O_4_, crystallizing in the $Fd\bar{3\;}m$ space group. The unit cell presents 32 oxygen atoms, which comprise 8 tetrahedral sites, usually occupied by the divalent cations, and 16 octahedral sites where the trivalent cation is positioned (see for example [1] and the references therein). The cation ordering in the described configuration is named “normal” and, since few years ago, was assumed to be characteristic of Zn-aluminates spinel [2]. Zinc, indeed, tends to form covalent bonds (with sp^3^ orbital hybridation) leading to an energy stabilization excess of this ion in the tetrahedral coordination [3]. However, a small degree of cations disorder (assumed as the amount of aluminium in the tetrahedral site, called “inversion” and quantified as the “degree of the inversion”) was already observed by Cooley and Reed in 1972 [4] and then confirmed by other authors in both synthetic and natural samples [5,6,7,8,9,10]. For example, Mathur and co-workers reported a degree of inversion of about 30% for nanocrystalline gahnite calcined at temperature lower than 900 °C [6]. Nanocrystals of gahnite attract much attention due to their use as semiconductors. The nanoparticles present a conduction band with explicit and specific energy levels that generate the so-called blue shift in the absorption edge as the particle size is decreased [6]. In addition, the gahnite optical bandgap makes this material transparent for light with wavelengths longer than 320 nm [11]; thus, this spinel can be useful as ultraviolet (UV) reflective coatings [6,12,13]. These optical properties were demonstrated to be linked to structural defect (i.e., inversion grade, oxygen vacancies and Zn^2+^ ions in interstitial positions) depending on the synthesis method [14].

Even though the structure of the spinel oxides is very well known, together with the variations of cation ordering (inversion degree) with temperature, the nanospinels present variations in the structure that are connected to the peculiar properties due to their size. Gahnite, which is a spinel with a medium degree of inversion, may be considered a model system for the study of structural variations at the atomic scale in spinel oxides, when the particle size reaches very small dimensions (just a few unit cells). For these reasons, it is crucial to have a complete and clear picture of the structure of this material when its particle size is very small, as well as when the particles grow, in order to understand where the limit between nanostructured and microstructured stands in this particular structure. In this work, we present a detailed structural analysis of nanocrystalline gahnite powders (2 nm) by means of traditional Rietveld and, for the first time, by Pair Distribution Function methods (PDF) [14,15,16,17]. Usually, material properties (especially for the nanostructured ones) are more related to the local than to the average structure [18,19,20,21]: structural studies at the local scale are then crucial for a complete material description. The original gahnite powders were also: i) treated with different calcination temperatures, in order to make them grow to about 10 nm size and ii) heated *in situ* to evaluate the influence of thermal treatment on structural evolution. Transmission Electron Microscopy (TEM) analysis were performed on all the investigated samples. 

## 2. Materials and Methods

### 2.1. Sample Synthesis

Samples were synthesized with the hydrothermal method proposed by Chen et al. [22]. Zinc and aluminum nitrates (10 and 30 mmol respectively) were firstly mixed, then 100 mmol of HMTA ((CH_2_)_6_N_4_), used as the basic source, was added drop by drop under magnetic stirring. The obtained solution was dissolved in 80 ml of distilled H_2_O and then transferred into a 100-ml Teflon-lined stainless steel autoclave, which was further heated at 120 °C for 24 hours. Resulting powders were washed with water and absolute ethanol, filtered and consequently dried. An initial calcination process was performed at 430 °C for 3 hours. Two further samples (named *gahnite-800 °C* and *gahnite-900 °C*) were obtained from the original sample (named *nanogahnite*) by thermal treatments at 800 °C and 900 °C.

### 2.2. Data Collection

Traditional X-ray powder diffraction data at room temperature were collected at ID31 high resolution beamline (currently ID22), European Synchrotron Radiation Facility (ESRF), Grenoble, France [23]. Diffracted intensities were measured by a bank of nine detectors, each preceded by a Si 111 analyser crystal with a wavelength set at 0.39996 Å. The sample *nanogahnite* was investigated also *in situ* as a function of the temperature between 140 °C to 940 °C using a cold-nitrogen-gas blower (Oxford Cryosystems, Long Hanborough, UK) sample environment.

Total scattering data of sample *nanogahnite* was measured at ID11, European Synchrotron Radiation Facility, Grenoble, France [24]. Wavelength was set at 0.20629 Å, collection was performed by a FReLoN camera (ESRF, Grenoble, France) recording 15 diffraction images with an exposure time of 3 seconds. Images were then merged together. 

Total scattering data collection of *gahnite-800 °C* and *gahnite-900 °C* was performed at ID15B, European Synchrotron Radiation Facility, Grenoble, France. Wavelength was set at 0.142155 Å; data collection was performed using a flat panel (40 × 40 cm) PerkinElmer detector (Perkin Elmer, Massachusetts, United States), with 20 individual images with an exposure time of 5 seconds. Images were then merged together.

### 2.3. Data Treatment

The average structure analysis was performed by means of Rietveld method using GSAS package [25] and EXPGUI interface [26]. The bibliographic structure of Cooley and Reed [4] was applied as starting model. No phase transition due to the reduced material particle size was observed. Profile fitting was performed between 3.7 and 35° 2*θ*. Chebyshev polynomial with 13 coefficients was used to model background. The peak profile was refined using a Thomson-modified pseudo-Voigt function, setting the peak cut-off at 0.1% of the peak maximum. Cell parameters were obtained for all the investigated samples, while structural analysis was not performed for *nanogahnite* and *nanogahnite* heated in situ from 140 °C to 460 °C due to the overwhelming peak broadening. For the two calcined samples (i.e., *gahnite-800 °C* and *gahnite-900 °C*) and *nanogahnite* heated *in situ* from 500 °C to 940 °C, isotropic thermal parameters of atoms belonging to the same chemical species were constrained to be equal. The zinc and aluminium occupancy values in both tetrahedral and octahedral sites were refined, as well as the oxygen atoms positions. However, constraints were imposed on Zn and Al occupancy factor in order to maintain the initial stoichiometry (i.e., the correct molar ratio of the two cations). 

Pair distribution function analysis was obtained using PDFgetX3 software [27]. Qmax was fixed at 25 (Å^−1^) for *nanogahnite* sample and at 30 (Å^−1^) for the calcinated ones, the background was subtracted and the scale value was set at 1. The meaning of these parameters is reported elsewhere [27,28]. In order to reduce noise in the low r region, rpoly was assigned a value of 1.4 Å. Local structural analyses were performed using PDFgui software [29]. The Qdamp parameter, obtained analysing a standard material (LaB_6_ NIST SRM 660b), was fixed at 0.08 for the data collected at ID15B, while at 0.06 for that collected at ID11.

### 2.4. TEM Analysis

TEM analysis was performed at the Department of Earth Science, University of Milan, using a FEI TECNAI F20 Field Emission Gun (FEG) transmission electron microscope. Instrument is equipped with *S*-Twin lens that lead a resolution of 0.24 nm. The imaging system is composed by one tv rate 626 Gatan and one slow scan 794 Gatan CCD cameras (Gatan, Peasanton, CA, United States). The system operates with an accelerating voltage of 200 kV. Investigated samples were prepared dispersing the powder in ethanol and ultrasonicating. The suspension (~ 5 µL) was deposited on a carbon-coated Cu grid.

## 3. Results

### 3.1. Average Structure

**Figure 1:**
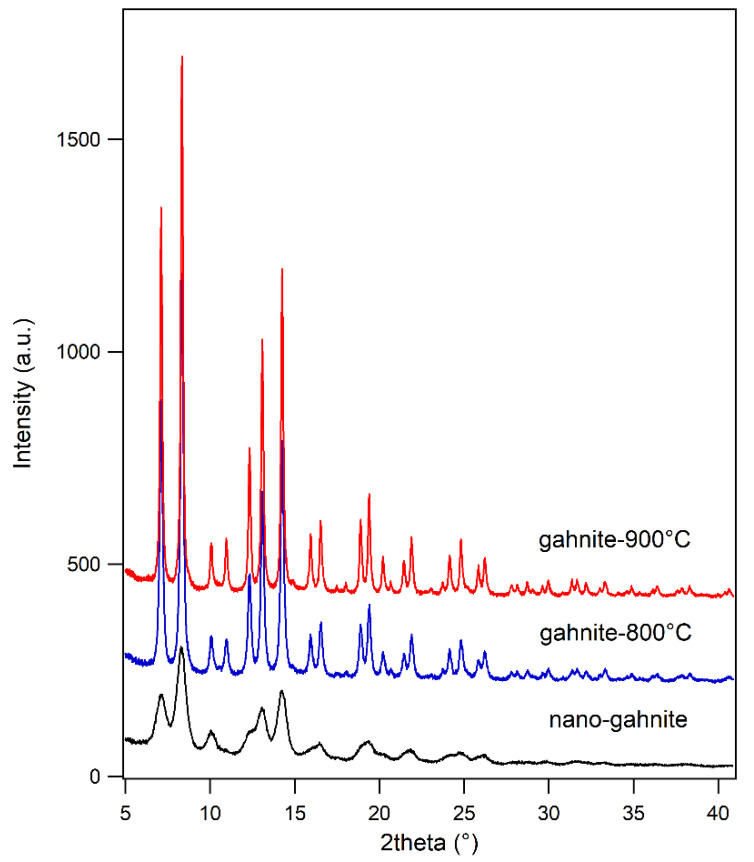
Diffraction patterns related to *nanogahnite*, *gahnite-800 °C* and *gahnite-900 °C* are reported.

Figure 1 shows the evolution of the diffraction patterns as a function of calcination temperature. Thermal treatment clearly influences the size of the samples as demonstrated by the decrease in peak broadening. The increasing calcination temperature also affects the peak positions and thus the cell parameters. Obtained values are 8.0858(7) Å, 8.0721(1) Å, 8.0736(1) Å respectively for *nanogahnite*, *gahnite-800 °C* and *gahnite-900 °C*. We have also to notice that the very large peak broadening of sample *nanogahnite* can lead to an error on cell parameters determination larger than that obtained by the minimization routine used for the refinements. Regarding the two calcined samples, the temperature influences the cell evolution slightly increasing the cell parameters.

Structural analysis (see Table 1, Table 2 and  Table S1, Figures S1 and S2 in the Supplementary Information) performed on the two calcined samples demonstrates a decreasing value of the degree of inversion (i.e., the occupancy factor of Al1, multiplied by 100, in the tables), as a function of calcination temperature.

Starting from the bibliographic data of 30% [6], likely ascribable also to the original *nanogahnite*, the occupancy of aluminium atoms into the tetrahedral site decreases from ~13% to ~10% for the calcination at 800 °C and 900 °C respectively. Consequently, due to the difference in size between Zn and Al ions)+, we observe a volumetric expansion of the tetrahedral site with an increasing distance between the centre site and oxygen atoms from 1.906(2) Å to 1.93104(2) Å.

**Figure 2:**
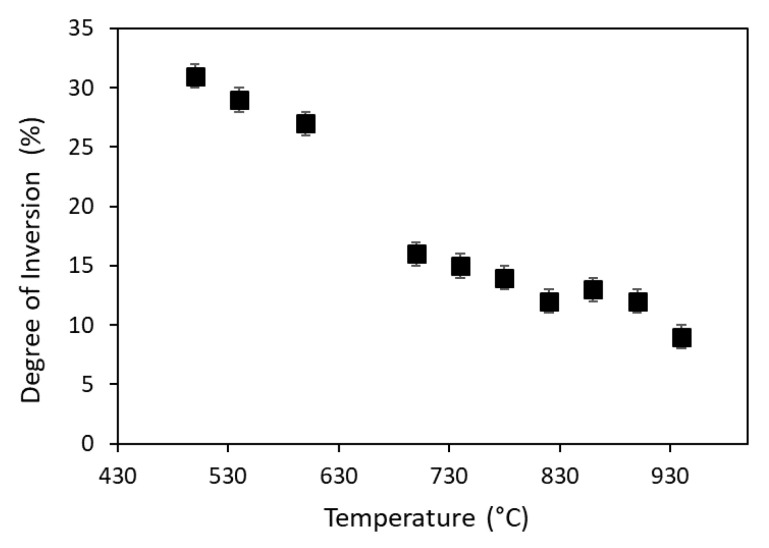
Degree of inversion of *nanogahnite* heated *in situ*, results are available only between 500 °C and 940 °C.

The degree of inversion can be seen also for the sample *nanogahnite* heated *in situ* (structural details are reported in the Table S2 of the Supporting Information as well as diffraction patterns in Figure S3) as shown in Figure 2. 

The initial value of 31(1)% reported for the sample heated at 500 °C is in perfect agreement with bibliography [6]. After an initial slow decrease between 500 °C and 540 °C, the percentage of aluminium in the tetrahedral site decreases faster, down to 16(1)% at 700 °C. The smallest value (9(1)%) is obtained at 940 °C. 

The work of Cornu and coworkers [14] indicates a dependence of the inversion grade from the temperature treatment, rather than from the used synthesis method (i.e., Pechini and co-precipitation). Our results extend this consideration also for the hydrothermal synthesis method. Indeed, our sample present a high initial amount of Al^3+^ in the tetrahedral site, that decreases as a function of the temperature. 

### 3.2. TEM and PDF Analysis: Grain Size Determination

**Figure 3:**
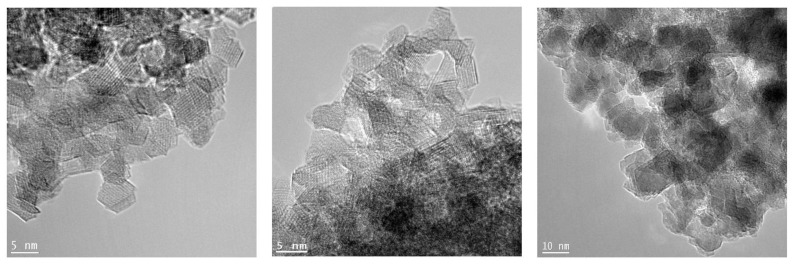
TEM images of *nanogahnite* (left panel), *gahnite-800 °C* (central panel) and *gahnite-900 °C* (right panel).

The particle size of nanosamples was calculated by means of PDF technique [19] (Table 3): results demonstrate an increase as a function of the calcination temperature. The rising particle size is confirmed by TEM analysis (Figure 3): comparison between the two techniques is reported in Table 3. The grain size was evaluated on different TEM images, and about 50–60 grains were measured and averaged to the value in Table 3. TEM images demonstrates a good crystallinity in the grains, no defect seems to affect samples at the medium scale, as the crystallographic planes are perfectly visible and well defined.

These observations suggest just little deviations of the local structure compared to the average one. 

### 3.3. Local Structure

**Figure 4:**
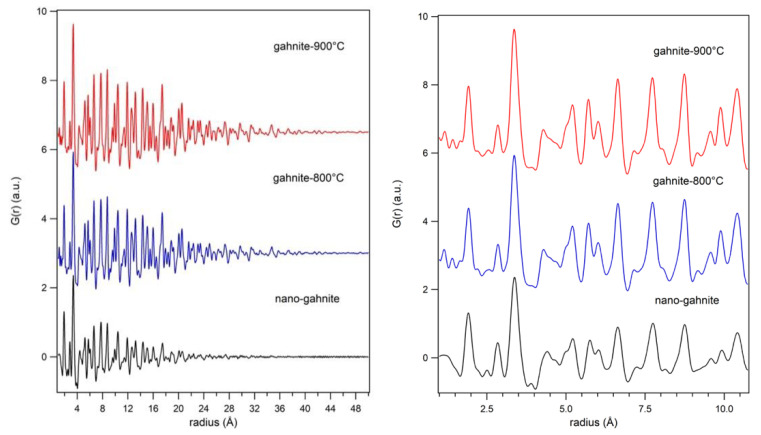
PDF related to *nanogahnite, gahnite-800 °C* and *gahnite-900 °C* are reported in two different radius ranges.

Figure 4 shows the PDF related to the investigated samples. As we can observe in Figure 4 (left panel), the difference in the decrease of the signal with r is proportional the different particle size of the investigated nanoparticles (see also previous section). The reduced particle size is at the basis of the local disorder of our samples. Indeed, between 1 and 10.75 Å, the structural models obtained at the average scale, even if refined, do not match satisfactory with the observed PDF. This misfit appears rather large for *nanogahnite*, decreasing in the other two samples, with a reasonable dependence on the particle size.

**Figure 5:**
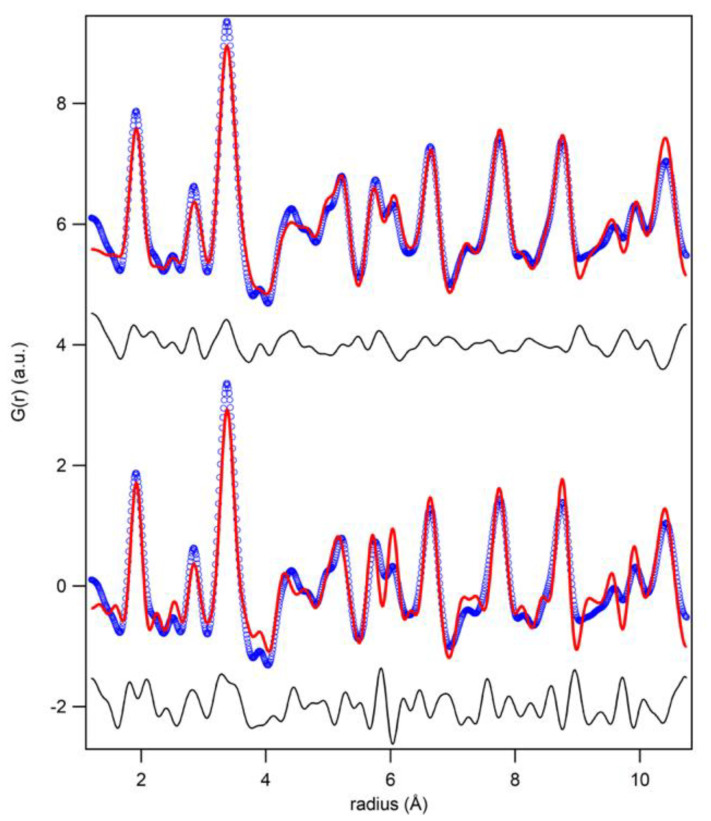
PDF refinement of the local range of the sample *nanogahnite* with the crystallographic structure (lower part) and with the proposed model (upper part).

Therefore, we identify in this range (Figure 5) the local structure. The lower part of the figure shows the fit for *nanogahnite* with the average structure found with the Rietveld method. In order to improve the fit of the local structure, a new structural model is proposed (the fit of the proposed model can be seen in Figure 5, upper part). Cell parameters are refined, isotropic thermal parameters are constrained to be equal for the same atomic species and then refined, while the occupancy factor of cations are kept fixed at the value obtained in the average structure. Oxygen atoms, originally positioned in (*xxx)* site, were displaced in a generic position (*xyz)* (changing the occupancy in order to maintain the correct stoichiometry, as site multiplicity changes) and the new coordinates were refined. Results obtained are reported in Table 4 and in Figure 5.

The comparison between average and local structure reveals slightly different cell parameters. Both *gahnite-800 °C* and *gahnite-900 °C* show a reduction of the local cell in comparison to the average one. Cell parameters of *nanogahnite*, instead, increase; this is not unusual in nanometric sample (as already reported in perovskite [30]), but, on the other hand, we have also to consider the average cell parameter as affected by a big uncertainty. 

There is still a misfit in the *nanogahnite* sample, recorded by its larger Rw, and visible in Figure 5, upper part. The goodness of fit parameter decreases from 22.9% for *nanogahnite* to 14.8% for gahnite-900 °C, showing that the model fits better for the sample with the larger grains. 

**Figure 6:**
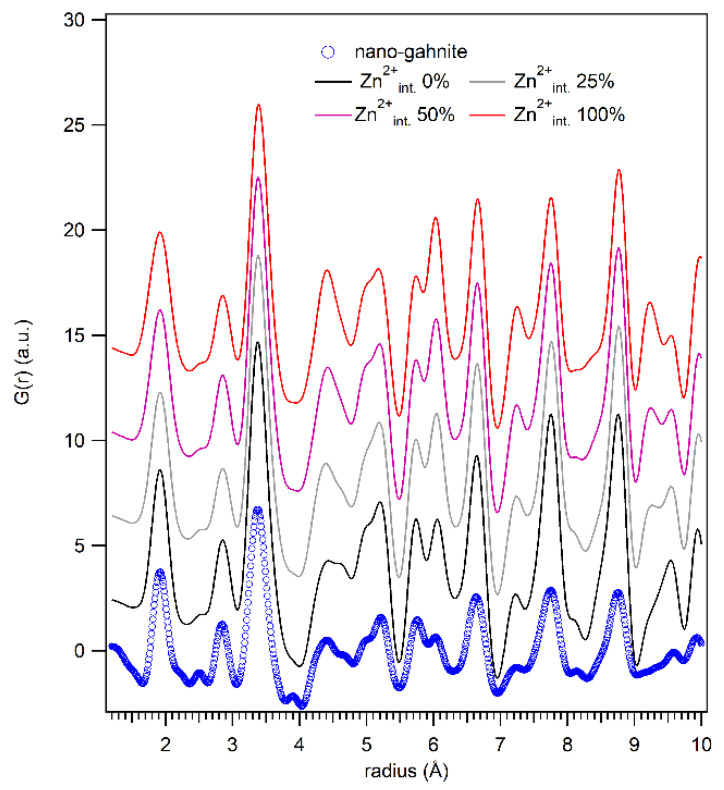
Partial PDF calculated from the refined structure obtained from the fit of the local range are reported against the data of *nanogahnite*. Each calculation was performed taking into account a hypothetical increasing value of Zn interstitial atoms (Zn^2+^_int._) expressed as a percentage of the total Zn atoms in the octahedral site.

In the work of Cornu and coworkers [14] the optical properties of *nanogahnite* (i.e., blue emission at 460 nm) was suggested to be linked to the presence of interstitial Zn^2+^, positioned in (*000)*, resulting from the inversion degree of the spinel. Considering the high Rw value obtained for the *nanogahnite,* we have to consider a possible shift of Zn atoms in interstitial positions. From the comparison of the calculated partial PDF (Figure 6), with an increasing value of the interstitial Zn^2+^, we can deduce that the ratio of the two peaks at 4.4 and 4.6 Å is distinctive for the presence of this defects. In *nanogahnite* sample data, this doublet seems similar to that reported for zero Zn interstitial atoms or to that of 25%. Thus, we tested a structural refinement implementing the additional position for Zn atoms (*000*) and refining its occupancy. Refinements confirm the absence of atoms positioned in the interstitial octahedra. Therefore, the proposed model with the oxygen disorder results, however, the more reliable to fit the local structure. 

From the results obtained, we can observe that the oxygen coordinates, for the sample *nanogahnite*, remain very close to the crystallographic *(xxx)* position, while there is a significant difference among the three coordinates in the samples with the larger grain size. 

This is originated by the different size of Al and Zn cations and their inversion grade. We can indeed suppose that, the octahedra containing only Al can be described with an oxygen environment which is peculiar (i.e.distances and polyhedral distortion) for this cation. The same can be presumed for Zn atoms in the octahedra position. Therefore, when the two cations are mixed (inversion of the spinel) we have the contribution on the average signal of two different polyhedral distortions and oxygen positions. The high inversion grade of nanogahnite thus produces on average, an intermediate oxygen coordinates that remain all equal, while in the calcinated samples the low inversion grade allows to better observe the two distortions peculiar of the Zn-tetrahedra and Al-octahedra.

In this light, it is also interesting to check how (and how much) the polyhedra are distorted in the local structure, with respect to the crystallographic average structure. Table 5 shows the average bond length, the polyhedral volume, the distortion index in both the octahedra and the tetrahedra composing the structure. These values are calculated by means of Vesta software [31]. The octahedra, for example, result far less distorted in the *nanogahnite* than in the more crystalline ones, which also show a larger spread between the minimum and the maximum distances in the polyhedra. On average, the octahedra are smaller for the three samples studied here, with respect to the crystallographic structure of Cooley and Reed [4], while the tetrahedra are larger. This is partly due, as already discussed, to the substitution of Zn by Al, and their difference in size. However, the larger distortion index for the octahedra of *gahnite-800 °C* and *gahnite-900 °C,* with respect to those pertaining to the average structure, may be only partly justified by this as the distortion of the polyhedral is produced by the increase of the degree of freedom for the oxygens in the unit cell in the model. 

## 4. Conclusions

The present work gives a detailed structural picture of nanocrystalline gahnite powders. Results presented demonstrate that:

- The calcination temperature (up to 900 °C) induces an increase in the particle size, as proved by the peak broadening trend, and a decrease in the degree of inversion. This last effect is also observable with *in situ* data collection as a function of temperature.
- TEM analysis reveals the nanosize of the investigated materials increasing with the calcination temperature. In addition, images prove the good crystallinity of the sample, even in those powders smaller than 2 nm, and the lack of evident defects at the medium scale.
- The local structure shows some differences from the average structure, as expected. In particular, a new structural model was proposed, with more degree of freedom for the oxygen coordinates, which fits better with the PDF data. The goodness of fit improves with grain size, while the deviation from the ideal *(xxx)* oxygen coordinates increases.
- It is suggested that particular properties exhibit from nanocrystalline gahnite are more related to a disorder of the oxygen atoms resulting from the inversion grade of the spinel rather than from interstitial Zn^2+^.
- The polyhedral volume for octahedra decreases, with respect to the one for the crystallographic structure, while, at the same time, the tetrahedral volumes increase. The degree of distortion of the octahedra is much larger for the two more crystalline samples than for the nanosized one: this may be partly due to the exchange between two different sized cations (Zn^2+^ and Al^3+^) and to the enlarged degree of freedom for the oxygen coordinates.
- The goodness of fit for proposed model on the local scale increases with the sample crystallinity, showing the model, though with a larger distortion of the polyhedra, is more suitable for the samples with a larger particle size.

For this model system, whose crystal structure is shared by many interesting materials, it is important to understand the differences between the structure of crystalline and microcrystalline samples from the one of the real nanometric ones. The real importance of this paper is that the local structure of all our three nanometric samples is different from the average one, but it is clear from the considerations above that the real nanostructured sample is different from the other two (more crystalline) samples. Therefore, it can be said that real nanostructured samples have a crystal size smaller than 7 nm in this particular spinel.

## Figures and Tables

**Table 1:** Atomic coordinates, occupancy factors and isotropic thermal parameters for *gahnite-800 °C*.

Atom	x/*a*	y/*b*	z/*c*	Occupancy Factor	UISO (Å^2^)
**Zn1**	0.125	0.125	0.125	0.865(4)	0.0038(1)
**Zn2**	0.5	0.5	0.5	0.054(2)	0.0038(1)
**Al1**	0.125	0.125	0.125	0.135(4)	0.0058(3)
**Al2**	0.5	0.5	0.5	0.946(2)	0.0058(3)
**O**	0.2613(1)	0.2613(1)	0.2613(1)	1	0.0014(3)

**Table 2:** Atomic coordinates, occupancy factors and isotropic thermal parameters for *gahnite-900 °C*.

Atom	x/*a*	y/*b*	z/*c*	Occupancy Factor	UISO (Å^2^)
**Zn1**	0.125	0.125	0.125	0.895(3)	0.0039(1)
**Zn2**	0.5	0.5	0.5	0.038(2)	0.0039(1)
**Al1**	0.125	0.125	0.125	0.105(3)	0.0057(3)
**Al2**	0.5	0.5	0.5	0.962(2)	0.0057(3)
**O**	0.2631(1)	0.2631(1)	0.2631(1)	1	0.0016(4)

**Table 3:** Comparison of particle diameters obtained by PDF and TEM analysis.

	Gahnite	Gahnite-800 °C	Gahnite-900 °C
**PDF (** **Å)**	27(3)	62(9)	69(11)
**TEM (** **Å)**	~ 40	~ 70	~ 90

**Table 4:** Refined parameters obtained by PDF analysis in the range 1–10 Å.

	*Nanogahnite*	*Gahnite-800 °C*	*Gahnite-900 °C*
**Cell Parameter (Å)**	8.092(8)	8.069(7)	8.062(6)
**Zn Uiso (Å^2^)**	0.006(2)	0.003(1)	0.004(1)
**Al Uiso (Å^2^)**	0.006(2)	0.007(2)	0.006(2)
**O Uiso (Å^2^)**	0.008(5)	0.009(3)	0.009(3)
**O *x* coordinate**	0.265(3)	0.263(5)	0.262(4)
**O *y* coordinate**	0.265(3)	0.269(3)	0.269(3)
**O *z* coordinate**	0.263(4)	0.261(5)	0.262(4)
**Rw (%)**	22.9	16.3	14.8

**Table 5:** Polyhedral characteristics in the nanosamples, compared to those of the average structure (PDF refinements in the range 1–10 Å).

	Bibliographic Structure [4]	*Nanogahnite*	*Gahnite-800 °C*	*Gahnite-900 °C*
**Octahedra**				
Average bond length (Å)	1.9226	1.9141	1.9090	1.9073
Average polyhedral volume (Å^3^)	9.3092	9.3105	9.8684	9.7433
Distortion index	0	0.00397	0.01396	0.01397
Min bond length (Å)	1.9226	1.91	1.87	1.87
Max bond length (Å)	1.9226	1.93	1.94	1.93
Standard deviation	0	0.097	0.02985	0.028
**Tetrahedra**				
Average bond length (Å)	1.9332	1.9529	1.9479	1.9462
Average polyhedral volume (Å^3^)	3.7079	4.0416	4.3559	4.1721
Distortion index	0	0	0	0

## Supplementary Materials

The following are available online at https://www.mdpi.com/2079-4991/10/5/824/s1: Table S1. Details of structural refinement parameters of *gahnite-800°C* and *gahnite-900°C*, Figure S1. Observed (red dash marks) and calculated (green line) diffraction patterns and final difference curve (purple line) from *gahnite-800°C*, Figure S2. Observed (red dash marks) and calculated (green line) diffraction patterns and final difference curve (purple line) from *gahnite-900°C*, Figure S3. Diffraction patterns of *nano-gahnite* heated in situ, Table S2. Cell parameters, occupancy factor of Al in tetrahedral site, oxygen atomic coordinates, isotropic thermal parameters and Rw value of the refinements of *nano-gahnite* heated in situ.
