# Supplementary material for: Structural Study of Nano-Sized Gahnite (ZnAl2O4): From the Average to the Local Scale
Source: Nanomaterials (Basel). 2020 Apr 26;10(5):824. doi: 10.3390/nano10050824 (PMC7712502; doi:10.3390/nano10050824)
Supplement: Supplementary file 1 [file nanomaterials-10-00824-s001.zip › SupportingINFO_gahnite_DEF_GC.docx]

**SUPPORTING INFORMATION**

**Structural study of nano-sized gahnite (ZnAl_2_O_4_): from the average to the local scale**

Giorgia Confalonieri^*^, Nicola Rotiroti, Andrea Bernasconi^**^, Monica Dapiaggi

*Dipartimento di Scienze della Terra, Università degli Studi di Milano, Milano, Italy.*

**Currently: Dipartimento di Scienze Chimiche e Geologiche, Università di Modena e Reggio Emilia, Modena, Italy.*

***Currently:* *Ideal Standard International, Trichiana (BL), Italy.*

Table S1. Details of structural refinement parameters of *gahnite-800°C* and *gahnite-900°C*.

| Sample | Rwp (%) | Rp (%) | RF^2^ (%) | Nobs | Nvar |
| --- | --- | --- | --- | --- | --- |
| Gahnite-800°C | 6.13 | 4.79 | 3.34 | 94 | 23 |
| Gahnite-900°C | 6.93 | 5.34 | 3.56 | 173 | 23 |


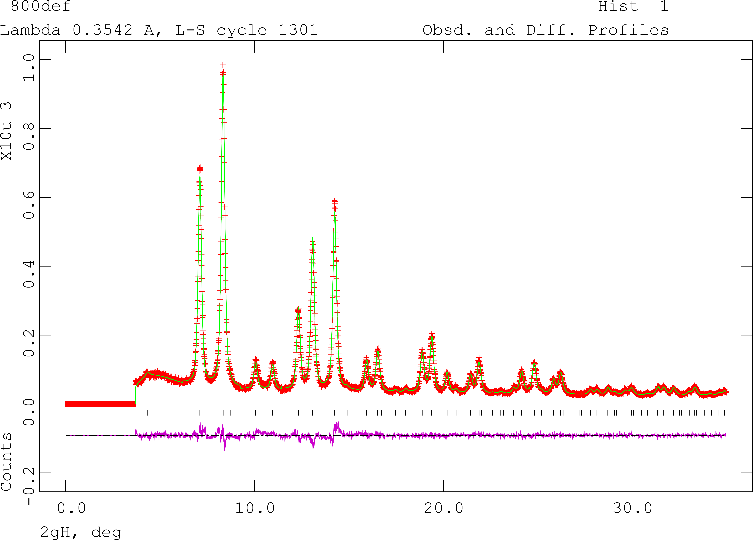


Figure S1. Observed (red dash marks) and calculated (green line) diffraction patterns and final difference curve (purple line) from *gahnite-800°C*.


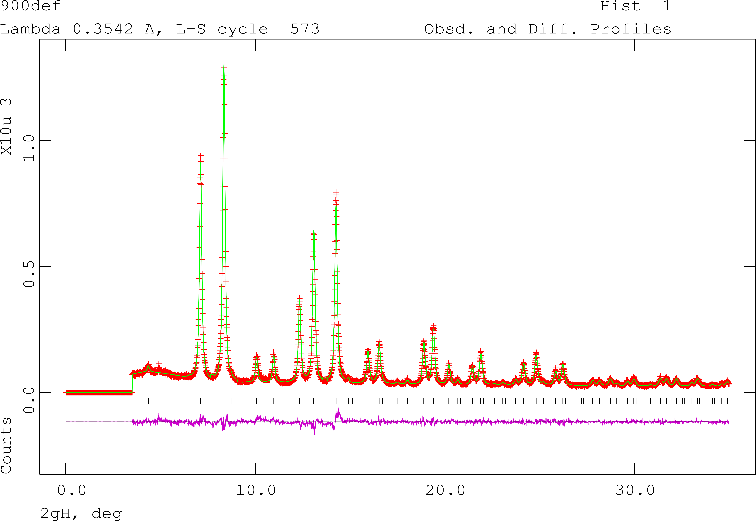


Figure S2. Observed (red dash marks) and calculated (green line) diffraction patterns and final difference curve (purple line) from *gahnite-900°C*.


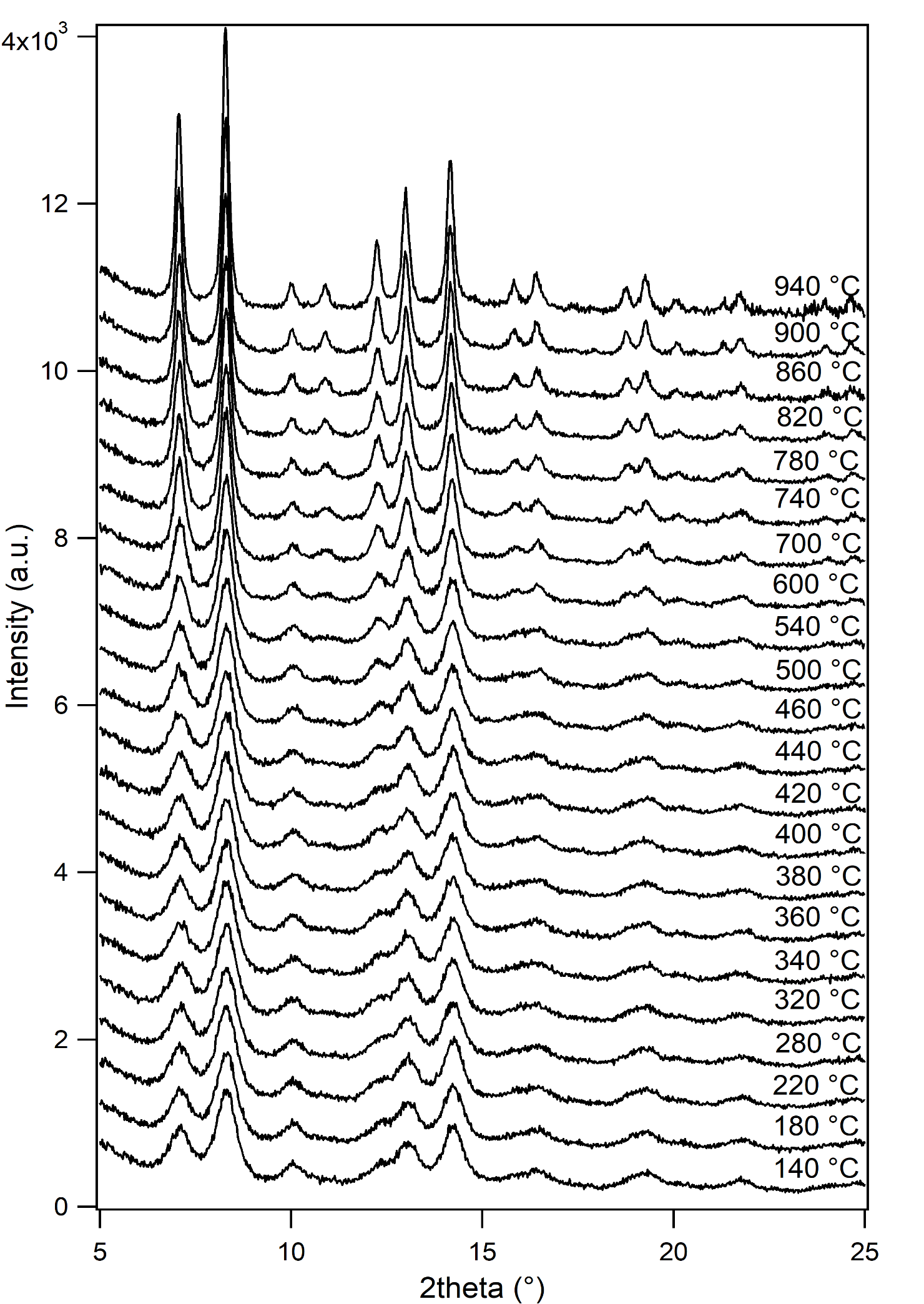


Figure S3. Diffraction patterns of *nano-gahnite* heated *in situ*.

Table S2. Cell parameters, occupancy factor of Al in tetrahedral site, oxygen atomic coordinates, isotropic thermal parameters and Rw value of the refinements of *nano-gahnite* heated in situ.

| Temperature | Cell Parameters (Å) | Al in Tetrahedral site | Oxygen coordinate | Uiso Zn (Å^2^) | Uiso Al (Å^2^) | Uiso O (Å^2^) | RF^2^ (%) |  |
| --- | --- | --- | --- | --- | --- | --- | --- | --- |
| 140°C | 8.093(1) |  |  |  |  |  |  |  |
| 180°C | 8.093(2) |  |  |  |  |  |  |  |
| 220°C | 8.094(1) |  |  |  |  |  |  |  |
| 280°C | 8.094(3) |  |  |  |  |  |  |  |
| 320°C | 8.100(1) |  |  |  |  |  |  |  |
| 340°C | 8.095(1) |  |  |  |  |  |  |  |
| 360°C | 8.098(3) |  |  |  |  |  |  |  |
| 380°C | 8.100(1) |  |  |  |  |  |  |  |
| 400°C | 8.099(1) |  |  |  |  |  |  |  |
| 420°C | 8.096(1) |  |  |  |  |  |  |  |
| 440°C | 8.099(2) |  |  |  |  |  |  |  |
| 460°C | 8.098(1) |  |  |  |  |  |  |  |
| 500°C | 8.100(3) | 0.31(1) | 0.2596(5) | 0.008(1) | 0.018(2) | 0.004(2) | 7.6 |  |
| 540°C | 8.101(1) | 0.29(1) | 0.2597(5) | 0.010(1) | 0.017(2) | 0.006(2) | 6.9 |  |
| 600°C | 8.1049(1) | 0.27(1) | 0.2612(5) | 0.012(1) | 0.016(1) | 0.004(2) | 6.6 |  |
| 700°C | 8.1107(6) | 0.16(1) | 0.2623(4) | 0.013(9) | 0.015(1) | 0.008(2) | 6.6 |  |
| 740°C | 8.1131(6) | 0.15(1) | 0.2620(4) | 0.012(9) | 0.017(1) | 0.006(2) | 7.8 |  |
| 780°C | 8.1151(5) | 0.14(1) | 0.2630(4) | 0.015(8) | 0.017(1) | 0.011(2) | 7.9 |  |
| 820°C | 8.1187(5) | 0.12(1) | 0.2633(4) | 0.0157(8) | 0.017(1) | 0.009(2) | 9.7 |  |
| 860°C | 8.1226(5) | 0.13(1) | 0.2634(4) | 0.016(10) | 0.011(2) | 0.004(2) | 10.8 |  |
| 900°C | 8.1272(4) | 0.12(1) | 0.2634(4) | 0.0146(7) | 0.010(1) | 0.007(1) | 8.01 |  |
| 940°C | 8.1311(4) | 0.09(1) | 0.2633(6) | 0.018(1) | 0.013(2) | 0.008(2) | 4.1 |  |
